# Synthetic control of a fitness tradeoff in yeast nitrogen metabolism

**DOI:** 10.1186/1754-1611-3-1

**Published:** 2009-01-02

**Authors:** Travis S Bayer, Kevin G Hoff, Chase L Beisel, Jack J Lee, Christina D Smolke

**Affiliations:** 1 Division of Chemistry and Chemical Engineering 1200 E. California Blvd., MC 210-41 California Institute of Technology Pasadena, CA 91125, USA

## Abstract

**Background:**

Microbial communities are involved in many processes relevant to industrial and medical biotechnology, such as the formation of biofilms, lignocellulosic degradation, and hydrogen production. The manipulation of synthetic and natural microbial communities and their underlying ecological parameters, such as fitness, evolvability, and variation, is an increasingly important area of research for synthetic biology.

**Results:**

Here, we explored how synthetic control of an endogenous circuit can be used to regulate a tradeoff between fitness in resource abundant and resource limited environments in a population of *Saccharomyces cerevisiae*. We found that noise in the expression of a key enzyme in ammonia assimilation, Gdh1p, mediated a tradeoff between growth in low nitrogen environments and stress resistance in high ammonia environments. We implemented synthetic control of an endogenous Gdh1p regulatory network to construct an engineered strain in which the fitness of the population was tunable in response to an exogenously-added small molecule across a range of ammonia environments.

**Conclusion:**

The ability to tune fitness and biological tradeoffs will be important components of future efforts to engineer microbial communities.

## Background

Many natural and man-made processes, such as lignocellulose digestion [1], wastewater treatment [2], environmental remediation [3], and biofilm formation [4] are mediated by consortia of microbes rather than a single organism. Often microbial consortia are composed of specialist strains that carry out individual metabolic reactions that benefit multiple community members, increase overall biochemical efficiency and buffer the community from environmental changes. In a recent example, a process involving two metabolic specialist strains of *Escherichia coli *was observed to efficiently convert xylose and glucose mixtures into fermentation products [5] more quickly than using a single generalist organism and adapted to changing concentrations of the two sugars by changing the relative abundance of each organism. The manipulation of existing microbial communities and the construction of synthetic communities will be increasingly important for engineering complex biological functions [6,7].

Synthetic biologists are beginning to design microbial consortia using bacterial quorum sensing. A recent study demonstrated how communicating populations of *E. coli *can act as an AND gate, exhibiting a gene expression response only when both populations are present [8]. Synthetic ecologies have been constructed where two types of bacteria act as 'predator' and 'prey', with each sensing the other by quorum sensing and dependent on the other for growth [9]. The ability to rationally engineer fitness in a given environment will advance the widespread use of microbial communities for performing biotechnologically-relevant processes.

Underlying the design of microbial consortia is the understanding of and ability to control fitness, communication, and ecological strategies [10,11] in a single organism. These tools and concepts can then be used to construct synthetic ecologies of interacting microbes useful in downstream engineering applications. Tradeoffs between fitness in different environments are well known in the ecology and engineering literature [12-15], and recent work has recapitulated such an ecological tradeoff by modulating the noise in expression of an antibiotic resistance gene [16]. Yeast populations driving the expression of an antibiotic resistance gene from noisy promoters were better able to survive challenge by antibiotics, but exhibited a fitness disadvantage in media without antibiotic. Such tradeoffs between stress resistance and fitness in stable environments have been observed in many classic ecological studies ranging from prokaryotes to metazoa to plants [10,13,17]. The ability to tune the performance of a population of cells for a given environment, including growth, adaptability, and stress resistance, will be useful in future engineering efforts.

Phenotypic variation between members of a population has been shown to be an important parameter in determining how organisms respond to biotic or abiotic environmental challenges [18]. Recent work has highlighted the prevalence of biological variability due to the fundamental limits of deterministic behavior at the cellular level [19-22]. Variability, or noise in gene expression, is a ubiquitous feature of the natural world and has been demonstrated to arise from the small number of molecules involved in cellular processes such as the levels of transcription factors, polymerases, and ribosomes [19]. Noise has been shown to be critical in several biological processes, including determination of competence in *Bacillus subtilis *[23,24], eye color vision development in *Drosophila melanogaster *[22], and viral latency in bacteriophages [25] and human pathogens [26].

Here, we examined the integration of synthetic regulation strategies with endogenous genetic networks to control 'ecological' parameters in a microbial population. Endogenous genetic networks dictate important ecological parameters, including fitness, phenotypic diversity [27], evolvability [28], and stress response, such that our ability to rationally manipulate these networks will be important to controlling more complex population- and consortia-level functions. We demonstrated that engineered strains differing only in the expression variability of an enzyme required for metabolizing ammonia, Gdh1p [29], displayed differences in fitness under 'normal' and stressful ammonia environments [30]. A strain exhibiting Gdh1p expression variability greater than the wildtype strain demonstrated increased resistance to ammonia stress, but lower fitness than wildtype at normal ammonia concentrations. A strain exhibiting lower variability in Gdh1p expression than wildtype displayed the opposite fitness trends – lower than wildtype resistance to ammonia stress and similar fitness to wildtype under normal ammonia concentrations. Finally, we constructed an engineered strain in which the fitness tradeoff was controlled by exogenous addition of a small molecule by placing the endogenous Gdh1p regulator, Dal80p [31], under the transcriptional control of a galactose-titratable promoter system [32]. Our results suggest that synthetic control of such fitness tradeoffs could be exploited to construct microbial populations and consortia with defined ecological behaviors.

## Results and Discussion

### Generation of mutants with different Gdh1p noise and abundance levels

**Figure 1:**
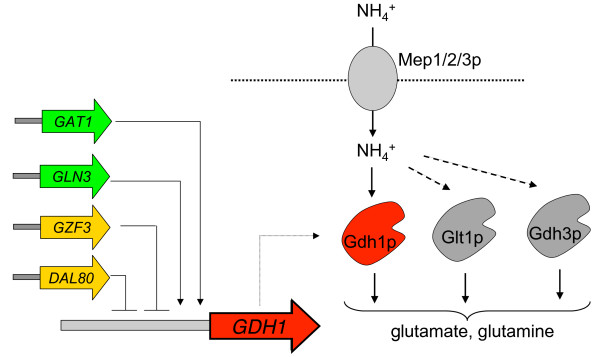
**Schematic of ammonia assimilation in *S. cerevisiae***. Ammonia is transported into the cell by three transporters, Mep1p, Mep2p, and Mep3p. Three enzymes, Gdh1p, Glt1p, and Gdh3p, convert ammonia to glutamate and glutamine. Gdh1p is responsible for the majority of ammonia assimilation. Gdh1p is regulated by four transcription factors, Gat1p, Gln3p, Dal80p, and Gzf3p.

We selected the ammonia metabolism of *S. cerevisiae *as a model system for engineering control over fitness tradeoffs. Ammonia is one of the preferred sources of nitrogen for yeast. Uptake of ammonia is performed by three transporters: two high affinity permeases, Mep1p and Mep2p, and one low affinity but high capacity permease, Mep3p [29]. Ammonia is used to either perform reductive amination of 2-ketoglutarate to form glutamate, the source of 80% of cellular nitrogen, or to synthesize glutamine from glutamate, which accounts for the other 20% [29]. These reactions are performed by two NADPH-dependent glutamate dehydrogenases, Gdh1p and Gdh3p, and a glutamate synthase, Glt1p. Gdh1p provides the primary route of ammonia assimilation under aerobic conditions and is regulated by at least four transcription factors, Gat1p, Gln3p, Gzf3p, and Dal80p (Fig. 1).

The toxic effects of ammonia have been well documented in animals and plants [33]; however, recent work by Hess *et al *demonstrated that yeast are also susceptible to ammonia toxicity [30]. Yeast possess mechanisms to excrete excess nitrogen in the form of amino acids, a rudimentary form of ammonia detoxification analogous to urea in mammals. The authors also demonstrated that ammonia toxicity is increased under low potassium ion levels, potentially due to the ability of ammonia ions to enter the cell via potassium channels. Therefore, while ammonia is an essential source of cellular nitrogen, environmental conditions, such as excess ammonia or low potassium, can be toxic to the cells. This paradoxical nature of ammonia (essential but toxic) provides a promising experimental system to examine the synthetic regulation of a fitness tradeoff between resource abundant and resource limited environments.

**Figure 2:**
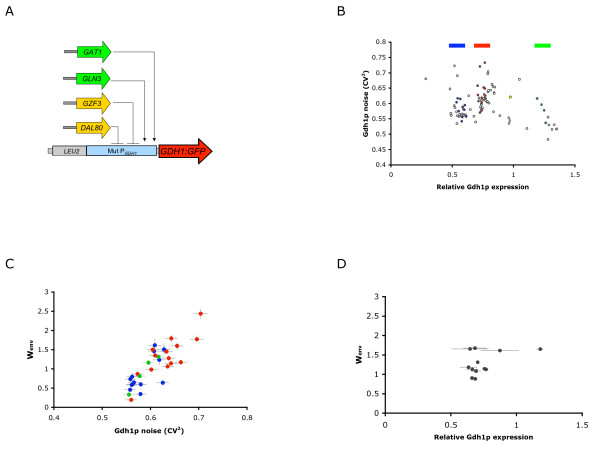
**Characterization of mutant strains with different Gdh1p noise and abundance profiles indicates that fitness in different ammonia conditions correlates with noise in Gdh1p expression**. (A) Schematic of the genetic construct for building the *GDH1 *promoter mutant library. A region 500 nt upstream of the *GDH1 *coding sequence was amplified by mutagenic PCR, assembled with a selectable *LEU2 *marker, and reintegrated to construct a mutant library. (B) A random set of clones from the *GDH1 *promoter mutant library exhibit different values for Gdh1p mean abundance and noise. Noise is calculated as the square of the coefficient of variation (CV^2^). Sets of mutants with low, wildtype, and high mean abundances are indicated as blue, red, and green, respectively. Wildtype is indicated in yellow. All errors are within 5% of the reported values. (C) Relative fitness in high and low ammonia concentrations correlates with noise in Gdh1p expression (correlation coefficient = 0.83, R^2 ^= 0.69). Relative fitness for select constant abundance-different noise mutant sets is reported as W_env_, or the ratio of fitness in 556 mM ammonia to fitness in 17 mM ammonia using the competition fitness assays. The wildtype relative fitness is equal to 1. Low Gdh1p noise is correlated with high fitness in ammonia-poor conditions and low fitness in ammonia-rich conditions (W_env _< 1). High Gdh1p noise is correlated with low fitness in ammonia-poor conditions and high fitness in ammonia-rich conditions (W_env _> 1). (D) Relative fitness in high and low ammonia concentrations does not correlate with Gdh1p abundance (correlation coefficient = 0.079, R^2 ^= 0.0062). Relative fitness (W_env_) for a set of *GDH1 *promoter mutants with similar Gdh1p noise values (approximate CV^2 ^= 0.65) and different abundance values is reported.

We implemented synthetic transcriptional control to examine the effects of changing the expression profile of a key enzyme in the ammonia assimilation pathway on the fitness tradeoff exhibited by the population under different ammonia concentrations. Previous studies have shown that mutations in promoter regions can contribute to both abundance and noise profiles in the expression of a downstream gene [34]. Therefore, we generated a set of Gdh1p expression mutants by random mutagenesis of the *GDH1 *promoter in a strain harboring a *GDH1:GFP *fusion (Fig. 2a). We examined the distribution of noise and mean abundance in Gdh1p expression for a set of randomly selected clones from the mutant library through flow cytometry. The clones exhibited a range of abundance and noise levels in Gdh1p (Fig. 2b), such that sets of strains were identified with constant abundance but different noise levels and constant noise but different abundance levels. Although we noted a slight correlation between noise and abundance, which has been observed in other studies of gene expression variability [21,35], we selected sets of mutants exhibiting different abundance levels for further characterization.

### Noise in Gdh1p and not abundance correlates with fitness across different ammonia environments

We used a previously reported direct competition assay [13] to examine the population fitness of the *GDH1 *promoter mutant clones under different ammonia concentrations. The relative contributions of noise and abundance in Gdh1p levels to the fitness tradeoff were determined by examining the fitness of sets of strains exhibiting constant abundance but different noise levels and constant noise but different abundance levels. To represent the relative contributions of fitness and stress resistance, we measured fitness via direct competition with a reference strain at both 17 and 556 mM ammonia. We defined a fitness term, W_env_, as the ratio of fitness at the higher ammonia concentration to fitness at the lower ammonia concentration, with W_env _for a wildtype strain containing the endogenous Gdh1p promoter equal to 1. Thus, clones with W_env _values greater than 1 exhibit enhanced growth at high ammonia versus low ammonia concentrations, while clones with W_env _values lower than 1 exhibit higher growth at low ammonia than high ammonia concentrations. Constant abundance-varying noise mutant sets with low, medium, and high abundance Gdh1p expression exhibited variations in W_env _that correlated with noise values (Fig. 2c). In contrast, W_env _did not show any correlation with abundance values under constant noise values (Fig. 2d). Our results demonstrate a correlation between Gdh1p noise and a fitness tradeoff across varying ammonia environments.

### A strain exhibiting higher variation in Gdh1p levels exhibits greater resistance to ammonia stress

**Figure 3:**
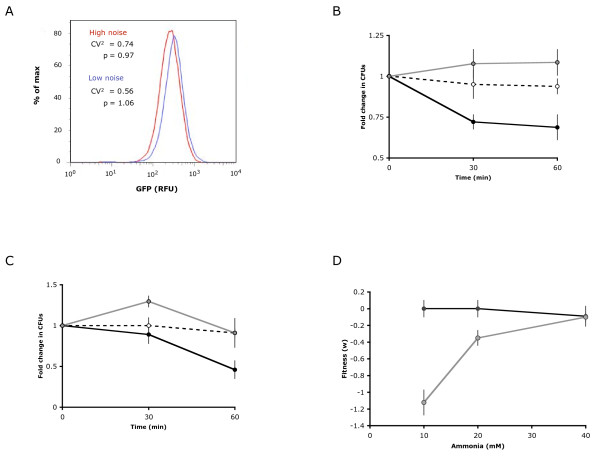
**Variation in Gdh1p expression provides different growth trends in high and low ammonia environments**. (A) Flow cytometry histograms of two *GDH1 *promoter mutants exhibiting different noise and similar abundance profiles in Gdh1p as the wildtype strain. The 'high noise' mutant has a square of the coefficient of variation (σ^2^/p^2^) of 0.74 (20% higher than wildtype) and the 'low noise' mutant has a σ^2^/p^2 ^of 0.56 (10% lower than wildtype). (B) The high noise mutant exhibits greater resistance to ammonia stress under high ammonia concentrations. The fold change in CFUs is reported at different time points following exposure to high ammonia (600 mM) media using the plate-based fitness assays. Dashed line, wildtype; gray line, high noise strain; black line, low noise strain. (C) The high noise mutant exhibits greater delayed toxicity to ammonia stress under high ammonia and low potassium concentrations. The fold change in CFUs is reported at different time points following exposure to high ammonia (600 mM) and low potassium (17 mM) media. Dashed line, wildtype; gray line, high noise strain; black line, low noise strain. (D) The low noise mutant exhibits greater fitness in low ammonia environments. Fitness for the high and low noise strains were measured across a range of low ammonia concentrations using the competition fitness assays. Fitness is reported as the natural log of the change in frequency over the growth period relative to the wildtype strain. Black circles, low noise strain; gray circles, high noise strain.

We selected two mutants from the *GDH1 *promoter library exhibiting similar abundances and different noise levels in Gdh1p expression to further examine the effects of Gdh1p noise values on the tradeoff between population growth in normal nitrogen environments and stress resistance in high ammonia environments. The 'high noise' mutant displayed 20% higher noise than the wildtype strain, while the 'low noise' mutant displayed 10% lower noise than the wildtype strain in minimal media with 40 mM ammonia (Fig. 3a). Noise remained unchanged when the mutants were grown in a range of ammonia concentrations (data not shown). We examined resistance to ammonia toxicity by transferring cultures growing in exponential phase to minimal media with high concentrations of ammonia (600 mM) and assaying the change in viable cell counts (measured as colony forming units, CFUs) by plating the cultures on rich media after a short amount of time (30 or 60 minutes; < 1 cell cycle) to observed initial fitness responses rather than long-term metabolic adaptation to ammonia stress. Wildtype cells displayed little change at 30 or 60 minutes after ammonia challenge (Fig. 3b). The high noise strain displayed a slight increase in CFUs after 30 minutes (~10%), whereas the low noise strain showed a marked decrease in CFUs after 30 minutes (~25%). Trends were similar in the low and high noise strains at 60 minutes after ammonia challenge. Results from this assay at later time points showed large variance between replicates, which necessitated a more sensitive assay of fitness as described below. Our results demonstrate that the high noise strain exhibits the greatest ability to adapt to stressful ammonia conditions, while the low noise strain exhibits the lowest resistance to toxic ammonia. These results suggest a correlation between noise in Gdh1p expression and stress resistance.

As previously indicated, ammonia toxicity is enhanced in environments with low amounts of potassium. To further examine the correlation between Gdh1p noise and stress resistance, we challenged the high and low noise strains in media with 600 mM ammonia and low potassium (17 mM), conditions at which ammonia is toxic to the cells. The high noise strain exhibited increases in CFUs at 30 minutes after ammonia challenge, but a decrease in viable cells at 60 minutes (Fig. 3c). In contrast, the low noise strain exhibited a slight loss of viable cells at 30 minutes after ammonia challenge that becomes more pronounced at 60 minutes. While it appeared that both strains were susceptible to the toxic effects of ammonia in low potassium media, the high noise strain displayed a significant lag period before the number of viable cells decreased. Such temporal differences in resistance may be critical in uncertain or fluctuating environments, where a high noise strain may be better able to buffer deleterious fluctuations in ammonia concentration.

### A strain exhibiting lower variation in Gdh1p levels exhibits greater fitness in physiological concentrations of ammonia

We also examined the effects of noise in Gdh1p expression on fitness under 'physiological' ammonia environments. Yeast are commonly grown in 40 mM ammonia in the laboratory, while ammonia concentrations in the natural yeast habitat may be lower [30]. Therefore, we measured population growth of the low and high noise strains across a range of low ammonia concentrations. The growth assays that examine increases in CFUs over time exhibited too much experimental error at these ammonia concentrations to reliably measure growth differences between the high and low noise strains and the wildtype strain. Therefore, we used the direct competition growth assay to measure fitness at multiple ammonia concentrations. The low noise strain exhibited similar fitness to wildtype at each assayed ammonia concentration (Fig. 3d). In contrast, the high noise strain exhibited significantly lower fitness than the wildtype and low noise strains at 10 mM and 20 mM ammonia, and similar fitness at 40 mM ammonia. Our results indicate that while the high noise strain exhibited increased resistance to ammonia stress, it also exhibited decreased fitness under lower ammonia concentrations, supporting the role of Gdh1p noise in affecting a tradeoff between stress resistance at high ammonia concentrations and fitness at low ammonia concentrations.

### Synthetic control of a Gdh1p regulator tunes noise in Gdh1p levels rather than abundance

**Figure 4:**
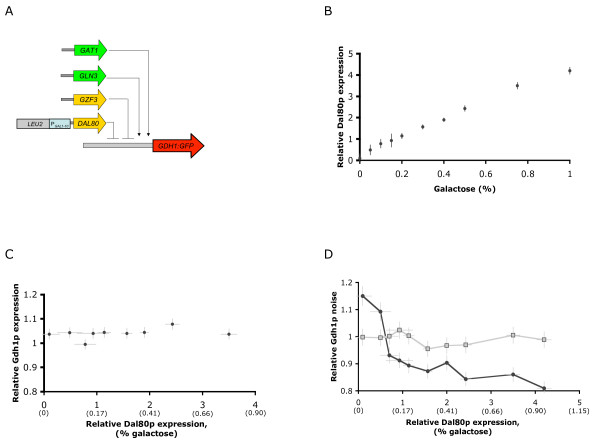
**Synthetic control of Dal80p levels tunes noise in Gdh1p expression**. (A) Schematic of the genetic construct for building the galactose-tunable Dal80p system. A region 500 nt upstream of the *DAL80 *coding region was replaced through homologous recombination with a construct encoding the *GAL1-10 *promoter and a selectable *LEU2 *marker. (B) *DAL80 *transcript levels vary linearly with exogenous galactose in the engineered strain. Relative *DAL80 *transcript levels were measured by qRT-PCR and are shown relative to wildtype *DAL80 *transcript levels. (C) Gdh1p abundance does not change as Dal80p levels change. The percent galactose added to the culture is shown in parentheses. (D) Noise in Gdh1p expression changes as Dal80p levels change. Populations of the engineered strain show higher noise at low Dal80p levels (low galactose concentrations) and lower noise with increasing Dal80p (increasing galactose concentrations). Percent galactose added to the culture is shown in parentheses.

Synthetic networks that enable regulation over fitness tradeoffs in a given environment in response to a small molecule that can be exogenously added to the system, will provide mechanisms through which to tune ecological strategies. Our results demonstrate that Gdh1p noise levels are correlated with the tradeoff in fitness across varying ammonia concentrations. We attempted to build titratable control over the observed fitness tradeoff by manipulating Gdh1p noise. We based our design on a synthetic control system that allows for the manipulation of Gdh1p noise levels in response to an exogenous small molecule. Alterations in the levels of transcriptional regulators have been shown to affect noise levels in regulated genes [16,34,36-38]. Gdh1p is regulated by at least four transcription factors: two activators, Gat1p and Gln3p, and two repressors, Gzf3p and Dal80p [31] (Fig. 1). We based our initial system design on the inducible control of the Gdh1p negative regulator, Dal80p. We replaced the endogenous *DAL80 *promoter with the *GAL1-10 *promoter through chromosomal integration in a strain background that harbored the *GDH1:GFP *fusion and a deletion in the galactose transporter Gal2p to achieve galactose-tunable control [32] of Dal80p (Fig. 4a). In our engineering strain, Dal80p expression levels changed linearly with galactose concentration as measured by quantitative real-time PCR, with most galactose concentrations inducing *DAL80 *levels greater than the wildtype *DAL80 *levels (Fig. 4b).

We measured the effects of varying Dal80p expression levels on the Gdh1p expression profile through flow cytometry analysis. Mean Gdh1p abundance did not change with varying Dal80p levels (Fig. 4c), indicating that combinatorial interactions at the *GDH1 *promoter or a complex regulatory network may be associated with this nitrogen assimilation pathway. However, the noise in Gdh1p expression decreased with increasing Dal80p levels (Fig. 4d). Specifically, in our engineered strain, low levels of Dal80p (under the absence of galactose) resulted in ~15% higher noise in Gdh1p expression than the wildtype strain, whereas high levels of Dal80p (in the presence of ~1% galactose) reduced noise to ~80% of the wildtype strain.

### Synthetic control of a Gdh1p regulator allows for tunable fitness across varying ammonia environments

**Figure 5:**
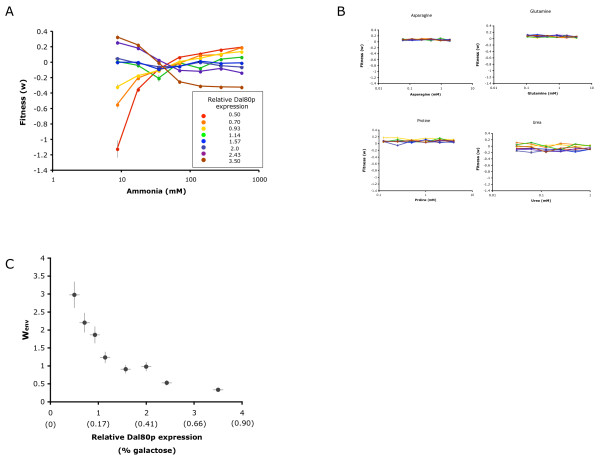
**Synthetic control of Dal80p allows for tuning of environment-dependent fitness**. (A) The fitness of the engineered strain displays varying trends with ammonia concentrations at different Dal80p expression levels. Dal80p expression levels were varied by altering the concentration of galactose in the media. Fitness was measured at the indicated ammonia concentrations using the competition fitness assays and is reported as in Figure 3d. (B) The fitness of the engineered strain does not change with varying concentrations of other nitrogen sources at different Dal80p expression levels. Dal80p expression levels were varied by altering the concentration of galactose in the media. Fitness was measured at the indicated ammonia concentrations using the competition fitness assays and is reported as in Figure 3d. (C) Relative fitness in high and low ammonia concentrations is tuned through the exogenous addition of galactose to the engineered strain. Relative fitness (W_env_) is reported as the ratio of fitness in 556 mM ammonia to fitness in 8.7 mM ammonia using the competition fitness assays. Low galactose concentrations tune the strain to exhibit greater fitness in ammonia-rich conditions than ammonia-poor conditions (W_env _> 1), whereas high galactose concentrations tune the strain to exhibit greater fitness in ammonia-poor conditions that ammonia-rich conditions (W_env _< 1).

We used the galactose-tunable control over Dal80p levels afforded by our engineered strain to measure population fitness at various Dal80p levels across a range of ammonia concentrations. We used the direct competition assay to measure fitness at ammonia concentrations spanning near growth limiting (17 mM) to near toxic conditions (600 mM) [30]. At low expression of Dal80p (lower than wildtype expression) the engineered strain displayed lower fitness than the wildtype strain at low ammonia concentrations, and higher fitness with increasing ammonia concentrations (Fig. 5a). In contrast, high Dal80p expression levels led to high relative fitness of the engineered strain at low ammonia concentrations and progressively lower fitness as ammonia concentrations increased. This fitness tradeoff is specific to ammonia as a nitrogen source (Fig. 5b), indicating that our synthetic control system tunes this fitness tradeoff for a specified environmental condition. Our engineered system is based on the modification of noise levels in a pathway enzyme (Gdh1p) through titration of the levels of a transcriptional regulator for that pathway enzyme (Dal80p). Our results demonstrate that our engineered strain can be altered to perform as a superior competitor at either high or low ammonia concentrations through the exogenous addition of a small molecule (Fig. 5c), demonstrating the synthetic tuning of a tradeoff between stress resistance and fitness in a stable environment.

## Conclusion

Variation between organisms in a population has long been recognized as an important parameter in predicting evolutionary dynamics. The past decade of research on the stochastic nature of gene expression has further highlighted the importance of variation on the functions of biological systems. Using a model of the interplay between variability and fitness tradeoffs [16], we discovered a similar tradeoff in yeast ammonia metabolism and achieved synthetic control of this tradeoff by manipulating noise through an endogenous regulatory circuit. We demonstrated that the level of noise in Gdh1p expression dictated the relative balance between resistance to toxic levels of ammonia and fitness in lower levels of ammonia. Furthermore, by examining the endogenous regulation of Gdh1p we discovered a convenient point in the circuit to regulate noise, and thus bring the fitness tradeoff under tunable control.

While our current work does not demonstrate a mechanism for stress resistance and fitness effects, similar studies in the literature may highlight routes for future investigation. Of particular interest is the mechanism by which increased noise in Gdh1p expression confers enhanced resistance to ammonia toxicity. One hypothesis is that in populations with larger Gdh1p distributions, the subset of the population with high Gdh1p expression is able to tolerate more ammonia by excreting more amino acids as described by Hess *et al *[30]. In this situation, at least a subset of the population would be able to survive temporary excesses of ammonia. The tradeoff between stress resistance and fitness at lower ammonia concentrations may be due to the energetic cost and deleterious effects of large fluctuations in protein expression, as has been observed in a previous bacterial study [35]. Such a mechanism may also explain why fitness tradeoffs were not observed with other nitrogen sources.

As potassium concentrations in the endogenous yeast habitat are likely lower than in laboratory conditions [30], the resistance to ammonia toxicity may be a significant contributor to survival of individuals and populations. Whether natural populations have taken advantage of modulating noise in enzyme expression in response to environments with excess ammonia, rather than manipulating amino acid excretion or other mechanisms, is an open and intriguing question, and is supported here by the observation that modulation of an endogenous transcriptional regulator modulates enzyme expression noise. Tuning adaptation to ammonia toxicity by synthetic manipulation of Gdh1p noise would result in populations that have low fitness in stable environments, but an enhanced ability to survive stressful periods. This phenotype is reminiscent of bacterial persistence, which has been shown to be driven by noise in gene expression [39]. As we continue to link cellular processes with ecological parameters we will gain new insight into evolutionary and ecological processes such as adaptation, variation, and evolvability. For example, recent computational studies have predicted that the design of regulatory networks are determined by the fitness benefits of regulating noise in a population [40]. In addition, the development of tools and concepts for manipulating ecological parameters will allow engineers to begin to more effectively build microbial consortia for potential applications in environmental remediation, energy, and therapeutics.

## Methods

### Strains and media

All manipulations were performed with the S288c background. Yeast were grown in synthetic complete media with the nitrogen source as specified. Primer sequences are provided in Additional file 1.

### Construction of mutant *GDH1 *promoter libraries

To construct mutant libraries of the *GDH1 *promoter, primers flanking 500 nucleotides upstream of the *GDH1 *coding region (1043500 – 1043050, chromosome XV) were used to amplify the fragment from yeast genomic DNA using KOD polymerase (Novagen) according to the manufacturer's instructions. The fragment was then diluted into mutagenic PCR buffer [41] (7 mM MgCl_2_, 0.5 mM MnCl_2_, 50 mM KCl, 10 mM Tris pH 8.3 with 1 mM dGTP, 0.2 mM dCTP, 0.2 mM dTTP, 0.2 mM dATP) and further amplified using Taq polymerase (Roche) according to the manufacturer's instructions. Separately, the leucine biosynthesis gene (*LEU2*) was amplified from pRS315 [42] using KOD polymerase. The *LEU2 *gene fragment and the promoter library were then ethanol precipitated, resuspended in water, and PCR assembled together through overlapping primer sequences. The resulting large fragment was then transformed into yeast strains using a standard lithium acetate procedure [43]. Transformants were selected in liquid synthetic complete leucine dropout media. The resulting library was grown to stationary phase and frozen in 15% glycerol at -80°C.

### Construction of the engineered Dal80p strain

Integration of the *GAL *promoter was performed by amplifying the *GAL1-10 *promoter sequence from pRS314-Gal [42] using KOD polymerase. This fragment was PCR assembled with the leucine biosynthesis gene (*LEU2*) from pRS315 [42] along with flanking homologous regions to the *DAL80 *upstream region (506000 – 504030 and 506500 – 506530 on chromosome XI) using KOD polymerase. The construct was transformed into yeast using a standard lithium acetate procedure and colonies were selected on synthetic complete leucine dropout agar plates. Integration was confirmed by colony PCR with primers flanking and internal to the integrated construct. Yeast DNA extraction was performed as previously described using the 'bust n' grab' method [44].

### Analysis of relative *DAL80 *transcript levels through quantitative RT-PCR

Cells were pelleted and frozen in liquid nitrogen. Pellets were resuspended in a 50 mM NaOAc (pH 5.2), 10 mM EDTA buffer. Cells were lysed by the addition of SDS to a final concentration of 1.6% and an equal volume of acid phenol. Solutions were kept at 65°C with intermittent vortexing for 10 min. After cooling on ice, the aqueous phase was extracted and further extraction was carried out with an equal volume of chloroform. RNA was further isolated and concentrated by use of RNeasy columns (Qiagen) according to manufacturer's instructions. Total RNA was quantified by OD_260 _readings. RNA samples were treated with DNase (Invitrogen) according to manufacturer's instructions. cDNA was synthesized using gene-specific primers and Superscript III reverse transcriptase (Invitrogen) according to the manufacturer's instructions. qRT-PCR was carried out on this cDNA using an iCycler iQ system (BioRAD). Samples were prepared using the iQ SYBR green supermix and primer pairs specific for different templates. Data were analyzed using the iCycler iQ software.

### Plate-based fitness assays

Yeast cells growing in exponential phase (OD_600_~0.5) were spun down, washed with sterile water, and resuspended in synthetic complete media with 600 mM ammonia. An aliquot of this culture was serially diluted (10-fold dilutions) and 50 μL of the dilutions were plated on YPD agar media. Cells were grown on the solid media for 2 days at 30°C and colonies were counted to measure the change in colony forming units (CFUs) over time.

### Liquid media competition fitness assays

Fitness was assayed by direct competition versus a common reference strain [13]. The competitor and reference strain constitutively express different fluorescent proteins (GFP and CFP, respectively) from the *ADH1 *promoter integrated into the chromosome. The frequency of competitor and reference strains were quantified before and after the growth period by counting the numbers of GFP expressing cells to non-GFP expressing cells. Fitness (w) of the competitor strain is reported as the natural log of the change in frequency of the strain during the competitive growth period versus the change in frequency of the reference strain over the same growth period:

**w **= ln (Δ_frequency _of competitor strain/Δ_frequency _of reference strain)

All competitor strains were derivatives of the S288C background, while the reference strain was derived from the W303 background. These strains showed different electronic volume versus side-scatter distributions that can also be used to quantify population numbers, in good agreement with the values obtained from fluorescent measurements. Equal amounts of competitor and reference strain were mixed and grown in indicated liquid media for 3 generations (approximately 6 hours). The frequency of competitor and reference strain were quantified before and after the growth period by counting the numbers of GFP expressing cells to non-GFP expressing cells by flow cytometry using a Quanta SC flow cytometer (Beckman Coulter) equipped with the MPL system. Samples were excited with a 488 nm laser and GFP fluorescence was detected with a 525 nm bandpass filter. A gate was set above the non-GFP expressing cells in the Quanta analysis software to partition fluorescent from non-fluorescent cells. Samples of only reference or competitor strains and serial dilutions of ratios of competitor to reference strains were run in parallel as controls. 5,000 events were collected per sample.

### Measurement of abundance and noise values through flow cytometry

Two gates were used to standardize each cell population for analysis using 'magnetic gating' in FlowJo flow cytometry analysis software (Tree Star, Inc.). The first gate isolated cells displaying regular morphology based on electronic volume and side-scatter, while the second gate removed non-fluorescent cells from the distribution. This gating method was compared against other methods previously described and the abundance and noise trends observed were consistent between methods [19,34]. Noise was calculated as the square of the coefficient of variation (σ^2^/p^2^) of the distribution [19]. Abundance was calculated as the mean of the distribution. 50,000 events were analyzed to calculate noise for each sample. Noise trends were similar when calculated as the coefficient of variation (σ/p) and the variance (σ^2^).

## Competing interests

The authors declare that they have no competing interests.

## Authors' contributions

TSB designed the experiments, performed the experiments, analyzed the data, and wrote the paper. KGH designed the experiments and analyzed the data. CLB designed the experiments, performed the experiments, and analyzed the data. CDS designed the experiments, analyzed the data, and wrote the paper.

## Supplementary Material

Additional file 1 **Supplementary Table 1.** Primer sequences used in constructing engineered strains. Click here for file
