# Supplementary material for: Synthetic control of a fitness tradeoff in yeast nitrogen metabolism
Source: J Biol Eng. 2009 Jan 2;3:1. doi: 10.1186/1754-1611-3-1 (PMC2631470; doi:10.1186/1754-1611-3-1)
Supplement: Additional file 1 — Supplementary Table 1. Primer sequences used in constructing engineered strains. [file 1754-1611-3-1-S1.pdf]

Supplementary Table 1. Primer sequences used in constructing engineered strains.

NAME	SEQUENCE
Gal/Dal80 F	CTGAGCGGGATATTGTTTATCTGCCGTCATGAGACGGTCACAGCTTGTCTG
Gal/Dal80 R	ATAATATGATATAATATAATGTAATATAGTCTAAAGGGAACAAAAGCTGGGTACG
Gdh1 mut.p fwd	CTTAGACCGCTCGGCCAAACAACACTCAGGTCATTATTCTCTAGTGTATCAGGCT
Gdh/leu mut rev	CTACATCCACTCAGGTCATTATTCTCTAGTCTGAGAGTGCACCATATCGACTACGTCG
Leu fwd	GTTGTTTGGCCGAGCGGTCTAAG
Gdh1 mut.p rev	TTTTGGTCTCCTAACTGTTATATTAGAATA
Qpcr Dal80.fwd	AAAAAGCTGAATAACAACAATGTGAA
Qpcr Dal80.rev	GGAACGGTTTCCTTTGGTTTTAA
